# Risk factors for lung metastasis at presentation with malignant primary osseous neoplasms: a population-based study

**DOI:** 10.1186/s13018-020-1571-5

**Published:** 2020-01-29

**Authors:** Lin Xie, Weibo Huang, Hongli Wang, Chaojun Zheng, Jianyuan Jiang

**Affiliations:** Department of Orthopedics, Huashan Hospital, Fudan University, 12 Mid-Wulumuqi Road, Shanghai, 200040 China

**Keywords:** Osseous neoplasms, Risk factor, Lung metastasis

## Abstract

**Background:**

Large population-based studies of risk factor for lung metastases at the presentation with primary osseous neoplasms are lacking and necessary. We aim to examine potential risk factors of lung metastases at presentation with primary osseous neoplasms using Surveillance, Epidemiology, and End Results (SEER) database tool.

**Methods:**

We collected patients diagnosed with primary osseous neoplasms between 2010 and 2015 from the SEER database. Patients were divided into two groups: patients with lung metastases or patients without lung metastases. Patient characteristics such as age, sex, race, tumor size, histologic types, histologic grade, and lung metastasis were collected. Univariate and multivariate logistic regression analyses were applied to determine which characteristics were risk factors for lung metastasis at diagnosis.

**Results:**

A total of 4459 patients were collected, and 507 patients had lung metastases at presentation. Data on age, race, gender, primary site, grade, tumor size, and histology types were enrolled into the multivariate logistic analysis. Higher grade (OR = 5.197, 95% CI 3.328 to 8.117), histology type (Ewing sarcoma: OR = 1.432, 95% CI 1.020 to 2.009; osteosarcoma: OR = 1.597, 95% CI, 1.073 to 2.377), and larger tumor size (≥ 5 cm: OR = 3.528, 95% CI 2.370 to 5.251) were associated with an increased risk of lung metastasis at presentation.

**Conclusion:**

Histology types (osteosarcoma and Ewing sarcoma) were related to a higher risk of lung metastases in primary osseous neoplasms patients. Patients with osteosarcoma and lager tumors or higher tumor grade were associated with higher possibility of lung metastases. Patients with Ewing sarcoma and larger tumors have more tendency of lung metastases. These patients are supposed to receive chest CT scans at the presentation with primary osseous neoplasms.

## Introduction

Primary osseous neoplasms were rare diseases with high mortality and affected patients of every age. Metastases were factors for worse prognosis for these patients, which might be a cause of death of these patients. Primary osseous neoplasms have metastatic potential to the lung, liver, and brain, and the lung is the main target. Although surgical amputation was often performed for patients with osteosarcoma before the advent of cancer chemotherapy, approximately 80% of patients still died of lung metastases [1, 2]. Among these patients, most of them have no obvious symptoms over a long period of time. In fact, according to previous literature, approximately 10–40% of patients had lung metastases at the presence of primary osseous neoplasms [3, 4]. Patients with lung metastasis were generally diagnosed by lung CT scan following diagnosis of primary osseous neoplasms. And the current treatment for patients with lung metastasis is complete surgical resection [5]. However, to the best of our knowledge, analysis of risk factors for lung metastasis in patients with primary osseous neoplasm has not been reported.

We used the Surveillance, Epidemiology, and End Results (SEER) Program database, which is commonly used for the analysis of rare cancer. The SEER database represents approximately 30% of the US population. Previous studies reported some social and clinical factors may be risk factors of lung metastases at presentation with malignant primary osseous neoplasms, but more researches are needed to reveal the associations.

The purpose of the present study was to examine the potential risk factors related to an increased rate of lung metastases at presentation with primary osseous neoplasms. A better understanding of the risk and the clinicopathological features of primary osseous neoplasm patients with lung metastases can help identify patients with high-risk factors and improve prognosis.

## Materials and methods

### Patient cohort

Patient information was accessed using the SEER database, which comprises 18 cancer registries and covers approximately 30% of the US population. The SEER database was accessed using SEER*Stat software (version 8.3.4).

The inclusion criteria were used as follows: (1) primary osseous neoplasms, (2) diagnosed between 2010 and 2015, (3) site limited to a bone only, and (4) diagnosis acquired in a living patient. The exclusion criteria were used as follows: unknown status of lung metastasis.

### Statistical analysis

The incorporated variables were compared between groups with the presence or absence of metastasis using chi-squared test. Univariate logistic analysis was used to select variables as possible risk factors associated with distant metastasis. Multivariate logistic analysis was then applied to determine the risk factors selected in the univariate analysis.

Chi-squared test, univariate logistic analysis, and multivariate logistic analysis were conducted via SPSS version 24. A two-sided *p* < 0.05 was considered statistically significant.

## Results

### Patient baseline characteristics

A total of 4459 primary osseous neoplasms patients diagnosed from 2010 to 2015 were collected from the SEER database. Patient characteristics are listed in Table 1. Of the 4459 patients with primary osseous neoplasms, 2530 (56.7%) were female and 1929 (43.3%) were male. A total of 1144 (25.7%) patients were diagnosed with low grade, and 1606 (36.0%) were diagnosed with high grade. Among these patients, 507 (11.4%) patients had lung metastases. Based on the chi-squared test between non-metastatic and metastatic patients, an increased rate of lung metastasis was found to be associated with younger age, extremity site, higher grade, lager tumor size, osteosarcoma, and Ewing sarcoma (Table 1).

**Table 1:** Characteristics of patients with malignant primary osseous neoplasms by lung metastasis

Characteristic	Total, *N* = 4459 No. (%)	Non-metastatic to lung, *N* = 3952 No. (%)	Metastatic to lung, *N* = 507 No. (%)	*P*
Age				< 0.001
< 30	1810 (40.6)	1521 (38.5)	289 (57.0)	
> 30	2649 (59.4)	2431 (61.5)	218 (43.0)	
Race				0.312
White	3667 (82.2)	3258 (82.4)	409 (80.7)	
Black	412 (9.2)	354 (9.0)	58 (11.4)	
Other^1^	348 (7.8)	312 (7.9)	36 (7.1)	
Unknown	32 (0.7)	28 (0.7)	4 (0.8)	
Gender				0.005
Male	2530 (56.7)	2213 (56.0)	317 (62.5)	
Female	1929 (43.3)	1739 (44.0)	190 (37.5)	
Primary site				< 0.001
Extremity	2273 (51.0)	1973 (49.9)	300 (59.2)	
Axial	2186 (49.0)	1979 (50.1)	207 (40.8)	
Grade				< 0.001
Low grade	1144 (25.7)	1117 (28.3)	27 (5.3)	
High grade	1606 (36.0)	1343 (34.0)	263 (51.9)	
Unknown	1709 (38.3)	1492 (37.8)	217 (42.8)	
Tumor size				< 0.001
< 5	1029 (23.1)	1000 (25.3)	29 (5.7)	
> 5	2667 (59.8)	2309 (58.4)	358 (70.6)	
Unknown	763 (17.1)	643 (16.3)	120 (23.7)	
Histology type				< 0.001
Osteosarcoma	1457 (32.7)	1225 (31.0)	232 (45.8)	
Ewing sarcoma	592 (13.3)	481 (12.2)	111 (21.9)	
Chondrosarcoma	1292 (29.0)	1223 (30.9)	69 (13.6)	
Squamous cell carcinoma	72 (1.6)	68 (1.7)	4 (0.8)	
Spindle cell sarcoma	54 (1.2)	45 (1.1)	9 (1.8)	
Chordoma	461 (10.3)	456 (11.5)	5 (1.0)	
Giant cell sarcoma	112 (2.5)	98 (2.5)	14 (2.8)	
Small cell sarcoma	5 (0.1)	3 (0.1)	2 (0.4)	
Epithelioid sarcoma	23 (0.5)	19 (0.4)	4 (0.8)	
Small round cell tumor	5 (0.1)	3 (0.1)	2 (0.4)	
Fibrosarcoma	62 (1.4)	54 (1.4)	8 (1.6)	
Adamantinoma	17 (0.4)	17 (0.4)	0 (0)	
Hemangiosarcoma	50 (1.1)	46 (1.2)	4 (0.8)	
Synovial sarcoma	16 (0.4)	12 (0.3)	4 (0.8)	
Leiomyosarcoma	25 (5.6)	24 (0.9)	1 (0.2)	
Other	216 (4.8)	178 (4.5)	38 (7.5)	

SEER 2010–2015

^1^Including American Indian/Alaska Native, Asian/Pacific Islander

### Risk factors for metastasis to lung at diagnosis

Data on age, race, gender, primary site, grade, and tumor size were enrolled into the univariate logistic regression analysis. Age, gender, primary site, grade, tumor size, and histology type were found to be associated with lung metastasis in the univariate analysis. After controlling for confounding variables using multivariate logistic analysis, gender, histology type, grade, and tumor size were identified as independent risk factors for lung metastasis at diagnosis, while age lost significance (Tables 2 and 3).

**Table 2:** Univariate and multivariate logistic regression analysis for patients with malignant primary osseous neoplasms by lung metastasis

Characteristic	Univariate analysis		Multivariate analysis	
	OR (95% CI)	*P*	OR (95% CI)	*P*
Age				
< 30	Reference		Reference	
> 30	0.472 (0.391–0.569)	< 0.001	0.902 (0.713–1.141)	0.391
Race				
White	Reference		NI	
Black	1.305 (0.971–1.755)	0.078		
Other^1^	0.919 (0.641–1.317)	0.646		
Unknown	1.138 (0.397–3.261)	0.810		
Gender				
Male	Reference		Reference	
Female	1.311 (1.084–1.586)	0.005	1.243 (1.020–1.516)	0.031
Primary site				
Extremity	Reference		Reference	
Axial	0.688 (0.570–0.830)	< 0.001	1.065 (0.860–1.319)	0.563
Grade				
Low grade	Reference		Reference	
High grade	5.346 (3.636–7.862)	< 0.001	5.197 (3.328–8.117)	< 0.001
Unknown	6.435 (4.239–9.770)	< 0.001	5.106 (3.223–8.088)	< 0.001
Tumor size				
< 5	Reference		Reference	
> 5	8.102 (5.409–12.134)	< 0.001	3.528 (2.370–5.251)	< 0.001
Unknown	6.017 (4.003–9.043)	< 0.001	4.346 (2.831–6.673)	< 0.001
Histology type				
Osteosarcoma	Reference		1.432 (1.020–2.009)	0.038
Ewing sarcoma	1.219 (0.949–1.564)	0.121	1.597 (1.073–2.377)	0.021
Chondrosarcoma	0.298 (0.225–0.394)	< 0.001	Reference	
Squamous cell carcinoma	0.311 (0.112–0.860)	0.024	1.233 (0.420–3.620)	0.704
Spindle cell sarcoma	1.054 (0.509–2.190)	0.884	1.749 (0.797–3.841)	0.163
Chordoma	0.058 (0.024–0.141)	< 0.001	0.102 (0.04–0.261)	< 0.001
Giant cell sarcoma	0.754 (0.423–1.344)	0.338	1.027 (0.545–1.934)	0.934
Small cell sarcoma	3.520 (0.585–21.183)	0.169	4.677 (0.700–31.234)	0.111
Epithelioid sarcoma	1.112 (0.375–3.297)	0.849	1.807 (0.576–5.668)	0.310
Small round cell tumor	3.520 (0.585–21.183)	0.169	5.216 (0.761–35.758)	0.093
Fibrosarcoma	0.782 (0.367–1.665)	0.524	1.505 (0.672–3.309)	0.321
Adamantinoma	NA	0.998	NA	0.998
Hemangiosarcoma	0.459 (0.164–1.288)	0.139	0.775 (0.263–2.287)	0.645
Synovial sarcoma	1.760 (0.563–5.505)	0.331	2.858 (0.852–9.591)	0.089
Leiomyosarcoma	0.220 (0.030–1.634)	0.139	0.386 (0.05–2.946)	0.358
Other	1.127 (0.773–1.644)	0.534	1.572 (0.992–2.493)	0.054

*OR* odds ratio, *CI* confidence interval, *NI* not included. *n* = 4459

^1^Including American Indian/Alaska Native, Asian/Pacific Islander

**Table 3:** Number (%) of patients with lung metastases according to histology type, tumor size, tumor grade, and gender

Characteristic	Number (%) of patients with lung metastases
Histology type	
Ewing sarcoma	111 (18.8)
Osteosarcoma	232 (15.9)
Chondrosarcoma	69 (5.3)
Chordoma	5 (1.1)
Tumor size	
< 5 cm	29 (2.8)
> 5 cm	358 (13.4)
Grade	
Low	27 (2.4)
High	263 (16.4)

Specifically, multivariate analysis demonstrated an increased risk of metastasis among male patients: OR = 1.243, 95% CI 1.020 to 1.516; patients with osteosarcoma: OR = 1.432, 95% CI 1.020 to 2.009; patients with Ewing sarcoma: OR = 1.597, 95% CI 1.073 to 2.377; patients with higher grade: OR = 5.197, 95% CI 3.328 to 8.117; and patients with a tumor larger than 5 cm: OR = 3.528, 95% CI 2.370 to 5.251.

### Risk factors for metastasis to lung at diagnosis of osteosarcoma

Data on age, race, gender, primary site, grade, and tumor size were enrolled into the univariate logistic regression analysis. Gender, grade, and tumor size were found to be associated with lung metastasis in the univariate analysis. After controlling for confounding variables using multivariate logistic analysis, grade and tumor size were identified as independent risk factors for lung metastasis at diagnosis, while gender lost significance. Specifically, multivariate analysis demonstrated an increased risk of metastasis among higher grade patients: OR = 4.861, 95% CI 1.948 to 12.131; and patients with lager tumor: OR = 3.432, 95% CI 1.715 to 6.867 (Table 4).

**Table 4:** Univariate and multivariate logistic regression analysis for patients with osteosarcoma by lung metastasis

Characteristic	Univariate analysis		Multivariate analysis	
	OR (95% CI)	*P*	OR (95% CI)	*P*
Age				
< 30	Reference		NI	
> 30	0.821 (0.608–1.108)	0.197		
Race				
White	Reference		NI	
Black	1.142 (0.779–1.673)	0.497		
Other^1^	1.043 (0.636–1.711)	0.867		
Unknown	1.083 (0.126–9.328)	0.942		
Gender				
Male	Reference		Reference	
Female	1.410 (1.057–1.881)	0.019	1.267 (0.945–1.698)	0.114
Primary site				
Extremity	Reference			
Axial	0.862 (0.623–1.192)	0.368	NI	
Grade				
Low grade	Reference		Reference	
High grade	5.769 (2.328–14.296)	0.000	4.861 (1.948–12.131)	0.001
Unknown	5.381 (2.106–13.751)	0.000	4.326 (1.681–11.136)	0.002
Tumor size				
< 5	Reference		Reference	
> 5	4.107 (2.064–8.171)	0.000	3.432 (1.715–6.867)	< 0.001
Unknown	6.281 (2.969–13.288)	0.000	5.470 (2.568–11.654)	< 0.001

*OR* odds ratio, *CI* confidence interval, *NI* not included. *n* = 1457

^1^Including American Indian/Alaska Native, Asian/Pacific Islander

### Risk factors for metastasis to the lung at diagnosis of Ewing sarcoma

Data on age, race, gender, primary site, grade, and tumor size were enrolled into the univariate logistic regression analysis. Tumor size was found to be associated with lung metastasis in the univariate analysis. After controlling for confounding variables using multivariate logistic analysis, tumor size was identified as an independent risk factor (OR = 5.213, 95% CI 2.052 to 13.245) for lung metastasis at diagnosis (Table 5).

**Table 5:** Univariate and multivariate logistic regression analysis for patients with Ewing sarcoma by lung metastasis

Characteristic	Univariate analysis		Multivariate analysis	
	OR (95% CI)	*P*	OR (95% CI)	*P*
Age				
< 30	Reference		NI	
> 30	0.942 (0.539–1.645)	0.833		
Race				
White	Reference		NI	
Black	1.509 (0.589–3.927)	0.399		
Other^1^	0.497 (0.192–0.288)	0.150		
Unknown	4.276 (0.595–30.728)	0.149		
Gender				
Male	Reference			
Female	1.205 (0.787–1.845)	0.391	NI	
Primary site				
Extremity	Reference			
Axial	1.232 (0.811–1.871)	0.329	NI	
Grade				
Low grade	Reference			
High grade	NA	0.999	NI	
Unknown	NA	0.999		
Tumor size				
< 5	Reference		Reference	
> 5	5.213 (2.052–13.245)	0.001	5.213 (2.052–13.245)	0.001
Unknown	5.820 (2.149–15.764)	0.001	5.820 (2.149–15.764)	0.001

*OR* odds ratio, *CI* confidence interval, *NI* not included. *n* = 592

^1^Including American Indian/Alaska Native, Asian/Pacific Islander

### Risk factors for metastasis to the lung at diagnosis of chondrosarcoma

Data on age, race, gender, primary site, grade, and tumor size were enrolled into the univariate logistic regression analysis. Gender, grade, and tumor size were found to be associated with lung metastasis in the univariate analysis. After controlling for confounding variables using multivariate logistic analysis, tumor grade was identified as an independent risk factor (OR = 6.204, 95% CI 3.483 to 11.048) for lung metastasis at diagnosis (Table 6), while gender and tumor size lost significance.

**Table 6:** Univariate and multivariate logistic regression analysis for patients with chondrosarcoma by lung metastasis

Characteristic	Univariate analysis		Multivariate analysis	
	OR (95% CI)	*P*	OR (95% CI)	*P*
Age				
< 30	Reference		NI	
> 30	3.216 (0.999–10.355)	0.05		
Race				
White	Reference		NI	
Black	1.038 (0.406–2.654)	0.938		
Other^1^	0.790 (0.242–2.586)	0.790		
Unknown	1.471 (0.188–11.500)	0.713		
Gender				
Male	Reference		Reference	
Female	1.683 (1.002–2.828)	0.049	1.502 (0.881–2.559)	0.135
Primary site				
Extremity	Reference		NI	
Axial	1.095 (0.674–1.782)	0.713		
Grade				
Low grade	Reference		Reference	
High grade	7.082 (4.018–12.484)	0.000	6.204 (3.483–11.048)	< 0.001
Unknown	3.277 (1.599–6.718)	0.001	3.137 (1.520–6.473)	0.002
Tumor size				
< 5	Reference		Reference	
> 5	2.992 (1.452–6.166)	0.003	2.038 (0.966–4.299)	0.062
Unknown	2.889 (1.195–6.984)	0.018	2.322 (0.944–5.711)	0.066

*OR* odds ratio, *CI* confidence interval, *NI* not included. *n* = 1292

^1^Including American Indian/Alaska Native, Asian/Pacific Islander

## Discussion

Lung metastases are of particular poor prognosis among patients with primary osseous neoplasms. It is assumed that approximately 10–40% of the patients have detectable lung metastases at diagnosis of malignant primary osseous neoplasms. In our study, analysis of the SEER database from 2010 to 2015 revealed that 11.4% cases of primary osseous neoplasms presented with lung metastases at the time of initial diagnosis. Osteosarcoma, Ewing sarcoma, larger tumors, and higher grade were associated with a higher risk of lung metastases in primary osseous neoplasm patients at diagnosis.

Primary osseous neoplasms are rare malignancies in the bone. Some of the more common malignant tumors include osteosarcoma, Ewing sarcoma, chondrosarcoma, and chordoma [6, 7]. Lung metastasis was the poor survival factor in these patients [8, 9]. Death from malignant bone tumors is usually the result of progressive lung metastasis with respiratory failure secondary to widespread disease [10, 11]. We found that the incidence of lung metastasis was 18.8% (Ewing sarcoma), 15.9% (osteosarcoma), 5.3% (chondrosarcoma), and 1.1% (chordoma). Ewing sarcoma is highly malignant with early metastasis to the lung and bone. Metastasis is commonly hematogenous and related to stemness [12]. Osteosarcoma is the most common primary malignant bone sarcoma and affects all ages. Previous studies report that 10–47% of osteosarcoma patients have lung metastasis at diagnosis [13]. Bielack et al. found that the incidence proportion of lung metastasis was 10.75% [14]. Kaste et al. reported that the incidence of lung metastases among patients with osteosarcoma was 15% [13]. Munajat et al. found that 33 patients (47%) had evidence of lung metastasis in a cohort of 70 patients with osteosarcoma [15]. Huang et al. found that the incidence of lung metastasis was 16.7% of patients with osteosarcoma [16]. In our study, we found osteosarcoma patients with larger tumor size or higher malignancy are risk factors for lung metastases. Chondrosarcoma is the second most frequent primary malignant bone sarcoma [17]. However, it is considered that metastasis is less common in patients with chondrosarcoma [17–19], while patients with chondrosarcoma and higher grade have more chance of lung metastases. Previous study reported that 11.2% of patients at 11 hospitals developed lung metastases after initial treatment of primary chondrosarcoma [20]. In our study, 5.3% (chondrosarcoma) has lung metastases. The rate of metastasis is related to histological tumor grade. Although Ewing sarcoma was a rare sarcoma type in younger age, in our study, Ewing sarcoma is the most common lung metastases sarcoma type. The rate of lung metastases is 18.8%. Patients with Ewing sarcoma and larger tumors have more chance of lung metastases.

Through the SEER database, we used large sample analysis to observe the risk factors of lung metastasis in malignant bone tumors. We determined that larger tumor size is an independent risk factor for lung metastasis at presentation. The larger the tumor size, the more lung metastases at diagnosis. Tumor size has been previously reported to be an independent prognostic factor for overall survival of patients with osteosarcoma and the occurrence of lung metastasis on plain radiographs or magnetic resonance imaging (MRI) [10]. Munajat et al. reported that larger tumors are more likely to correlate with lung metastasis in their cohort of 70 patients with osteosarcoma [15]. Changes in tumor size may be closely related to its biological behavior [21]. A large primary tumor is more likely to be associated with distant metastasis.

We also identified higher tumor grade as an independent risk factor for lung metastasis at diagnosis. Previous studies reported that 40–60% of patients of the high-grade sarcomas will develop lung metastases, of which 70–80% will have disease limited to lungs, likely through hematogenous spread [21]. Bjornsson et al. revealed that high grade (II or III) was significantly related to both increased risks of recurrence (36.2% of recurrence at 10 years vs. 15.2% for grade I; *p* < 0.001) and metastasis (29.3% of metastasis at 5 years vs. 4.6% for grade I; *p* < 0.001) [20]. Giuffrida et al. found that high-grade tumors had a hazard ratio of 3.4 for death when compared with low-grade tumors [22]. Song et al. found that higher grade was associated with an increased risk of metastasis at presentation, which demonstrated the high tendency to metastasize in high-grade tumors [23].

Several limitations should be taken into consideration in the present study. First, we did not include other potential risk factors such as pathologic fracture, which has proven to be a prognostic factor for survival. The reason was that the SEER database did not record these variables. In addition, specific location of the tumors or complete information regarding the size was not available in the SEER database. Finally, our results were analyzed based on retrospective cohort data; thus, prospective researches are needed for further validation.

## Conclusions

The present study identified risk factors for lung metastases in patients with primary osseous neoplasms. It would be helpful for clinicians to evaluate patients’ risk of lung metastasis and counsel patients regarding the possibility of lung metastasis at diagnosis. Moreover, our study could provide insights into clinicopathological factors which are related to the development of lung metastatic primary osseous neoplasms to diagnosis.

## Data Availability

The patients’ data were collected from SEER database.

## Abbreviations

CI: Confidence interval
MRI: Magnetic resonance imaging
NI: Not included
OR: Odds ratio
SEER: Surveillance, Epidemiology, and End Results
